# Influence of Antibiotics on Functionality and Viability of Liver Cells In Vitro

**DOI:** 10.3390/cimb44100317

**Published:** 2022-10-03

**Authors:** Sandra Doß, Corinne Blessing, Katharina Haller, Georg Richter, Martin Sauer

**Affiliations:** 1 Department Extracorporeal Therapy Systems (EXTHER), Fraunhofer Institute for Cell Therapy and Immunology, Schillingallee 68, 18057 Rostock, Germany; 2 Department of Anesthesiology and Intensive Care Medicine, Charité Campus Benjamin Franklin, Hindenburgdamm 30, 12203 Berlin, Germany; 3 Center for Anesthesiology and Intensive Care Medicine, Hospital of Magdeburg, Birkenallee 34, 39130 Magdeburg, Germany; 4 Department of Anesthesiology and Intensive Care Medicine, University Hospital of Rostock, Schillingallee 35, 18057 Rostock, Germany

**Keywords:** antibiotics, drug-induced liver injury, hepatotoxicity

## Abstract

(1) Antibiotics are an important weapon in the fight against serious bacterial infections and are considered a common cause of drug-induced liver injury (DILI). The hepatotoxicity of many drugs, including antibiotics, is poorly analyzed in human in vitro models. (2) A standardized assay with a human hepatoma cell line was used to test the hepatotoxicity of various concentrations (Cmax, 5× Cmax, and 10× Cmax) of antibiotics. In an ICU, the most frequently prescribed antibiotics, ampicillin, cefepime, cefuroxime, levofloxacin, linezolid, meropenem, rifampicin, tigecycline, and vancomycin, were incubated with HepG2/C3A cells for 6 days. Cell viability (XTT assay, LDH release, and vitality), albumin synthesis, and cytochrome 1A2 activity were determined in cells. (3) In vitro, vancomycin, rifampicin, and tigecycline showed moderate hepatotoxic potential. The antibiotics ampicillin, cefepime, cefuroxime, levofloxacin, linezolid, and meropenem were associated with mild hepatotoxic reactions in test cells incubated with the testes Cmax concentration. Rifampicin and cefuroxime showed significantly negative effects on the viability of test cells. (4) Further in vitro studies and global pharmacovigilance reports should be conducted to reveal underlying mechanism of the hepatotoxic action of vancomycin, rifampicin, tigecycline, and cefuroxime, as well as the clinical relevance of these findings.

## 1. Introduction

Infections are a common problem in the intensive care unit (ICU), mainly due to the severity of patient illness and the frequent use of invasive procedures. Recent data suggest that 51% of ICU patients are infected, and 71% receive antibiotics [1,2], with broad-spectrum antibiotics representing one of the most frequently administered treatments in the ICU [3]. Worldwide consumption of antibiotics has dramatically increased in recent decades [4].

Adverse drug-related events (ADEs) are more frequent and severe among critically ill patients as a result of their weakened immune systems. There is often the need for prophylactic calculated administration of antibiotics as a result of the patient’s critical condition; thus, symptoms indicative of infection may be treated immediately without a full diagnosis.

Generally, evaluation of the incidence of antibiotic-associated ADEs in hospitalized patients is unavailable. Shehab et al. conducted a retrospective analysis of ADEs among patients admitted to emergency departments at the Johns Hopkins Hospital (Baltimore, Maryland). The aim of the study was to estimate and compare the number and frequency of emergency department visits for drug-related side effects associated with systemic antibiotics in the US by event type, individual drug, and drug class. The results of the study show that nearly 20% of patients treated with antibiotics for at least 24 h experience adverse side effects [5]. Potentially life-threatening events associated with ADEs occur in 26% of ICU patients as compared to 11% in non-ICU patients (*p* < 0.001) [6]. It is estimated that antibiotics are prescribed ten times more frequently in intensive care units than in general wards, which significantly increases the risk of side effects [7]. Whereas drug interactions are more frequent in ICU patients, they also face a higher risk for drug accumulation due to organ failure, as well as higher sensitivity to drug response because of their unstable status, leading to severe complications and diverse forms of toxicity, including hepatotoxicity, neurological dysfunction, acute kidney injury (AKI), cardiopulmonary toxicity, and other organ disorders.

Antibiotics are the single largest class of agents that cause drug-induced liver injury (DILI) [8]. DILI can mimic and be confused with any liver injury; therefore, the injury phenotype is highly variable; it can be acute or chronic and is classified as either cholestatic, hepatocellular, or mixed injury [9]. This describes the type of histological and chemically determined liver cell damage in the laboratory and is calculated on the basis of the R quotient, i.e., the quotient of the serum value of alanine aminotransferase (ALT; x-fold increase above normal) and the serum value of alkaline phosphatase (AP; x-fold increase above normal) in relation to their upper limit of the normal range [10].

Antibiotic-induced hepatotoxicity is usually abnormal, momentary, unpredictable (depending on the pharmacology of the antibiotic), and mainly dose-independent. Rifampicin is an antibiotic belonging to the class of antituberculosis medications; it has been associated with major side effect, such as DILI [11]. Antibiotic-associated hepatotoxicity sometimes manifests as other liver diseases and presents nearly all the symptoms of acute and chronic liver injury, ranging from asymptomatic elevation of liver enzymes to fulminant hepatic failure. Treatment is therefore based on clinical notions and can involve the elimination of underlying causes. It sometimes depends on the elimination of competing etiologies, such as viral hepatitis, biliary diseases, or acute heart failure; however, the identification of real cholestatic liver dysfunction remains a challenge. A rare event is antibiotic-induced intrinsic DILI, which is characterized by a closed dose-dependent pathway and predictability. For instance, rifampicin can cause intrinsic DILI, as well as idiosyncratic DILI.

The frequency of severe antibiotic-related hepatotoxicity is low relative to the quantities prescribed annually. Population-based estimates suggest that <5 cases per 100,000 antibiotic prescriptions are associated with hepatotoxicity, which remains a major reason for withdrawal of approved drugs [12].

Nevertheless, it is important to understand that most antibiotics can cause idiosyncratic hepatotoxic reactions. Diagnosis of DILI remains a challenge; therefore, the development of reliable hepatotoxic biomarkers is in full progress. Several methods have been developed to facilitate the assessment of hepatotoxicity causality. These liver-specific causality assessment tools fall into three categories: (1) probabilistic approaches, (2) expert judgments, and (3) algorithms or scales [13,14]. Clinical studies are generally limited in terms of the identification of trends in hepatotoxicity, such that case reports of adverse drug reactions are the main source of toxicity data [15]. The current dominant strategies for testing DILI potential are based on in vivo animal models that do not efficiently predict the DILI potential in humans. Significant species-specific variation between rodents and humans, as well as genetic variability in humans, influences the conclusions made with respect to the clinical situation [16,17]. A recent analysis showed that 43% of human toxic effects were correctly predicted by rodent testing, whereas non-rodent inclusion increased to 63% [18]. For DILI research, the prospective collection of phenotypic information in corresponding registries and biological samples from identified DILI cases is currently the most valuable resource to reproduce the complexity of idiosyncratic DILI due to the lack of reliable animal models [19,20,21,22].

LiverTox (https://livertox.nih.gov (accessed on 22 August 2022)), a clinical research database that provides an overview of medications, includes a description of the pattern and history of liver damage, as well as case studies supported with lab data and a comprehensive list of references. However, the standard drug compendia and published reports on the potential hepatotoxicity of drugs vary widely. Therefore, it is recommended to routinely consult LiverTox to stay up-to-date on the latest DILI reports [23].

The purpose of this study is to examine often-used antibiotics in intensive care unit (ICU) settings for their hepatotoxic potential. An established and standardized hepatocyte biosensor was successfully employed for antibiotic assessment in immortalized human hepatocyte HepG2/C3A. These cells are characterized by their functional similarities to the human liver, as well as their physiological response to toxic insults and metabolic markers [24,25,26,27,28].

## 2. Materials and Methods

### 2.1. Cell Culture and Drug Treatment

Human hepatocellular carcinoma cells (HepG2/C3A; ATCC #CRL-10741) were cultured as adherent monolayers in Dulbecco’s Modified Eagle Medium (DMEM, GIBCO Life Technologies, Darmstadt, Germany) supplemented with 2 mM L-glutamine, 10% FBS (fetal bovine serum; Biochrome, Berlin), and 1% antibiotic solution (Penicillin G: 10.000 IE/mL/Streptomycin: 10 mg/mL; Biochrome, Berlin) and placed in a 37 °C, 5% CO_2_ humidified incubator. The passage number did not exceed 10, and cells were regularly subcultured every 2–3 days.

Depending on the specific established microtiter plate hepatotoxicity assay, HepG2/C3A cells were seeded at a density of 5 × 10^5^ cells/mL in 24-well tissue culture plates [26,27,28]. Cells were treated with seven antibiotics at varying concentrations: Ampicillin (430 µM, 2150 µM, and 4300 µM; ratiopharm, Ulm, Germany) [29], cefepime (210 µM, 1050 µM, and 2100 µM; MIP Pharma GmbH, Blieskastel, Germany), [30,31] cefuroxime (140 µM, 700 µM, and 1400 µM; Fresenius, Bod Homburg, Germany) [32,33], levofloxacin (18 µM, 90 µM, and 180 µM; Ebert, Ursensollen, Germany) [34], linezolid (44 µM, 220 µM, and 440 µM; betapharm, Augsburg, Germany) [35,36], meropenem (130 µM, 650 µM, and 1300 µM; Fresenius Kabi, Bad Homburg, Germany) [37], rifampicin (24 µM, 120 µM, and 240 µM; Riemser, Greifswald, Germany) [38], tigecycline (0.85 µM, 4.25 µM, and 8.5 µM; Pfizer, NY, USA) [39], and vancomycin (0.0069 µM, 0.0345 µM, and 0.069 µM; Ebert, Ursensollen, Germany) [40] for 2 × 3 days. The lowest concentrations of the various antibiotics, i.e., the mean plasma level after induction of I.V. therapy (Cmax), as well as the 5-times and 10-times concentrations of Cmax, were analyzed. Each concentration was tested in triplicate. Experiments were independently repeated five times. Whereas antibiotic-free medium or plasma served as a negative control, acetaminophen (APAP, 15.24 mM in medium; Sigma Aldrich, Seelze, Germany) was used as a positive control. The pH values (Radiometer, ABL, Willich, Germany) were measured in the cell culture supernatant either before treatment or 72 and 144 h after treatment.

### 2.2. Cell Proliferation and Death

A simple in vitro output measure of hepatotoxicity is cell death assessment. Cell death was calculated by lactate dehydrogenase and trypan blue exclusion assays ((0.4% (*w*/*v*); Sigma, Seelze, Germany). Lactate dehydrogenase (LDH) was released into the culture medium following the loss of membrane integrity as a result of cell death. After harvesting the supernatants, LDH activity was measured photometrically by detecting the change in absorbance at 340 nm (automated chemistry analyzer, Cobas Mira, Roche, Mannheim, Germany) according to the optimized standard method of the Deutsche Gesellschaft für Klinische Chemie (DGKC) [41]. On the other hand, trypan blue is a commonly used biological staining reagent that can detect the loss of cell membrane permeability; therefore, cells were manually counted using a Neubauer chamber and light microscopy to determine the cellular vitality (viable cells divided by the total number of cells).

### 2.3. Enzyme Activity Assays and Albumin Release

The XTT assay ((2,3-Bis-(2-Methoxy-4-Nitro-5-Sulfophenyl)-2H-Tetrazolium-5-Carboxanilide) 3-(4,5-di-methylthiazol-2-yl)-2,5-diphenyltetrazolium) is a colorimetric tetrazolium reduction assay (AppliChem GmbH, Darmstadt, Germany) used to quantify functional cell impairment. It is based on the measurement of mitochondrial enzyme activity in viable cells that reduce the tetrazolium derivative XTT. After 1 h of incubation, the optical density (OD) was measured with an ELISA reader (Anthos Reader 2001, Anthos Labtec Instruments, Wals-Siezenheim, Austria) at a test wavelength of 450 nm and a reference wavelength of 650 nm [42].

Metabolic activity in the form of ethoxyresorufin-O-deethylase activity (EROD, cytochrome P(450) 1A2) was quantified using a methodology previously established in our laboratory based on a method described by Donato et al. [27,43]. The induction reagent β-naphthoflavone (BNF) was briefly added to the hepatocytes 72 h before treatment and measurement. After 24 h of incubation with varying concentrations of antibiotics, EROD activity was quantified by the fluorometric detection of resorufin at excitation and emission wavelengths of 530 nm and 584 nm, respectively. Fluorescence was measured with a fluorescence multiwell plate reader (Clario Star, BMG Labtech, Ortenburg, Germany). Concentrations were estimated against a resorufin standard curve (0 pmol, 10 pmol, 20 pmol, 40 pmol, and 80 pmol).

The concentration of human albumin in the supernatants was determined nephelometrically from 0.2 mL cell culture medium or plasma (Immage 800, Beckman Coulter GmbH, Germany) [44].

### 2.4. Statistical Analysis

Non-normally distributed experimental data were analyzed and presented as median and 25th–75th percentiles with GraphPad Prism 5 (GraphPad Software, La Jolla, CA, USA). Kruskal–Wallis and Mann–Whitney non-parametric tests were used for pairwise comparisons of groups. Statistical significance was set at *p* < 0.05.

## 3. Results

### 3.1. Rifampicin and Cefuroxime Significantly Reduce Vitality and Cell Proliferation in a Dose-Dependent Manner

**Figure 1:**
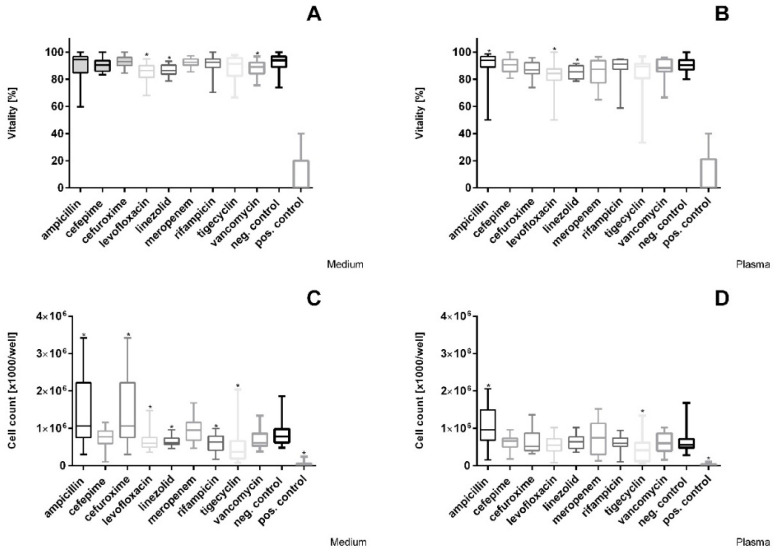
Representation of the Cmax concentration. Vitality (trypan blue staining) of HepG2/C3A cells after exposure to (**A**) antibiotics in medium and (**B**) antibiotics in plasma, as well as cell count of hepatocytes after exposure to (**C**) antibiotics in medium and (**D**) antibiotics in plasma at Cmax. Values represent median and 25th/75th percentile of three replicate wells from four different experiments. Significance between control and exposure groups is indicated by * *p* < 0.05.

The effects on cell proliferation and vitality varied significantly among the tested antibiotics. Compared to the negative control, vitality was significantly reduced after incubation with the Cmax concentrations of levofloxacin and linezolid in medium and plasma, whereas vancomycin was only decreased in medium (Figure 1A,B). The other antibiotics did not show significant changes in the medium or plasma.

**Figure 2:**
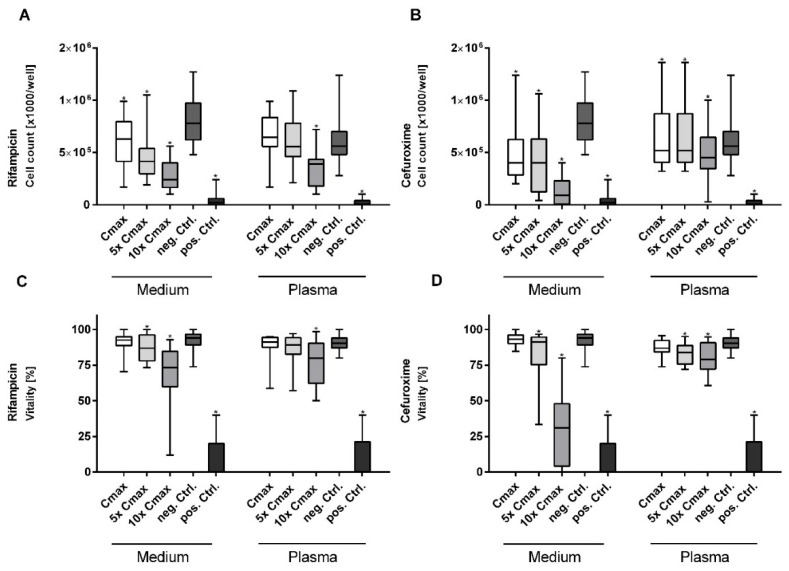
The upper graphs represent a dose-dependent decrease in vitality after hepatocytes treatment with (**A**) rifampicin and (**B**) cefuroxime with a stepwise increase in concentration. The lower graphs show a dose-dependent decrease in cell count after treatment of hepatocytes with (**C**) rifampicin and (**D**) cefuroxime with a stepwise increase in concentration. * denotes a significant decrease in cell count compared with the control group. *p* < 0.05 was considered statistically significant.

Whereas there was no effect observed at Cmax, cell vitality was significantly decreased at higher concentrations of rifampicin and cefuroxime, especially in medium (Figure 2C,D). For cefuroxime vitality was reduced to 32.24% at 10× Cmax, which was the strongest effect observed within among all tested antibiotics and close to the effect of the positive control (25%) (Figure 2A,B). The other antibiotics did not show a dose-dependent impairment of cell vitality.

A significant reduction in cell count compared to the negative control was detected after incubation with cefuroxime, levofloxacin, linezolid, rifampicin, and tigecycline at Cmax in medium; for tigecycline, a reduction was observed in medium, as well as in plasma (Figure 1C,D).

As the concentration was increased, a further decrease was observed for rifampicin and cefuroxime, principally in medium (Figure 2A,B). The other test substances showed no significant dose-dependent effects on cell proliferation.

### 3.2. Cefuroxime Causes the Highest Dose-Dependent Loss of Cell Integrity (LDH)

**Figure 3:**
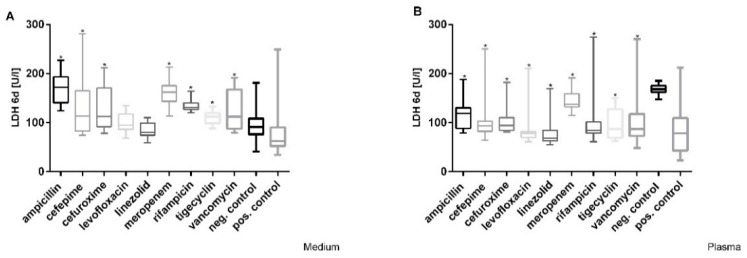
Effects of varying Cmax antibiotic concentrations on LDH release in (**A**) medium and (**B**) plasma in HepG2/C3A cells during 6 days of treatment. Values represent median and 25th/75th percentile of three replicate wells from four experiments. Significance difference between control and exposure groups is indicated by * *p* < 0.05.

A significant increase in lactate dehydrogenase (LDH) at Cmax was detected after incubation with ampicillin, cefepime, cefuroxime, meropenem, rifampicin, tigecycline, and vancomycin. In particular LDH values increased by more than 50% compared to the negative controls (91 U/L) after treatment with ampicillin, meropenem, and rifampicin at Cmax in medium (Figure 3A). The testing of Cmax concentrations of antibiotics in plasma showed that all LDH levels were significantly lower than in healthy plasma after 6 days (Figure 3B). Dose-dependent effects were only observed after incubation with cefuroxime in medium with a stepwise increase in concentration in cell culture supernatant (LDH values of 113–261 U/L) after 6 days; additionally, only cefuroxime showed a dose-dependent increase in LDH from 94 to 155 U/L at its highest concentration after 6 days in plasma.

### 3.3. Effects of Antibiotics on the Activity of Mitochondrial Dehydrogenase in Hepatocytes

**Figure 4:**
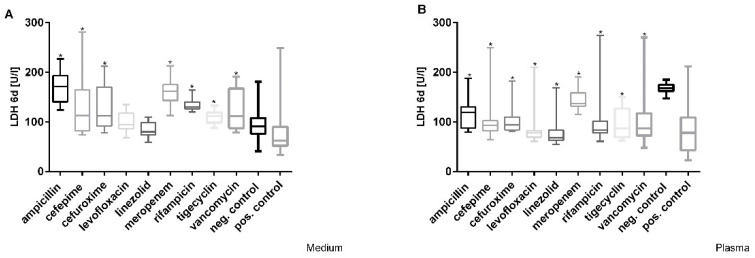
Metabolic activity for HepG2/C3A following 6 days of antibiotics exposure at Cmax quantitatively measured (**A**) in medium and (**B**) plasma using an XTT colorimetric assay. Values represent the median and 25th/75th percentile of three replicate wells from four experiments. Significance difference between the control and exposure groups is indicated by * *p* < 0.05.

The activity of mitochondrial dehydrogenases (XTT-test) of HepG2/C3A cells was significantly decreased at the Cmax of levofloxacin (median optical density (OD) 1.065), rifampicin (0.984), tigecycline (1.054), and vancomycin (1.079) compared with the negative control in medium (1.301), whereas it was significantly increased for ampicillin (1.523), cefepime (1.486), cefuroxime (1.55), meropenem (1.327), and linezolid (1.394) (Figure 4A). A negative dose-dependent effect on mitochondrial activity was observed only for cefepime (from 0.216 to 1.55).

Cells incubated in plasma showed increased activity of mitochondrial dehydrogenases after treatment with ampicillin (1.430), cefuroxime (1.674), linezolid (1.273), and rifampicin (1.382) at the Cmax concentration (Figure 4B). In contrast, cells treated with levofloxacin (1.002), meropenem (0.921), tigecycline (0.929), and vancomycin (1.096) showed a significant decrease in mitochondrial activity (cefepime: 1.221, negative control 1.2) at Cmax. Significant dose-dependent effects were not detected as a result of increasing plasma concentrations of the tested drugs.

### 3.4. Decrease in Cytochrome 1A2 Activity

**Figure 5:**
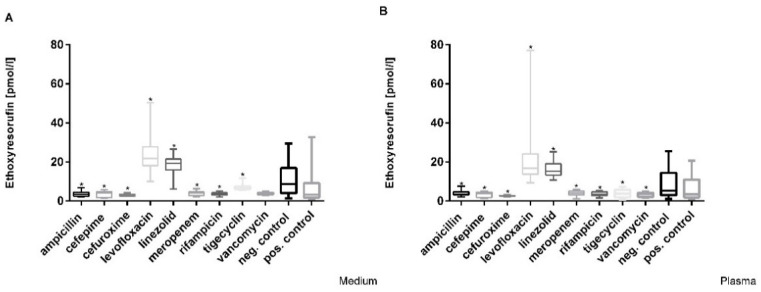
EROD induction in hepatocytes incubated at Cmax and exposed to (**A**) antibiotics in medium and (**B**) antibiotics in plasma. Values represent median and 25th/75th percentile of three replicate wells from four experiments. Significant difference between the control and exposure groups is indicated by * *p* < 0.05.

In the hepatocyte cultures, the control group showed an EROD activity level of around 8.7 pmol/L in medium and 5.3 pmol/L in plasma (Figure 5). A significant increase in EROD activity was observed after incubation with levofloxacin and linezolid (Cmax) in medium and plasma, whereas ampicillin, cefepime, cefuroxime, meropenem, rifampicin, tigecycline and vancomycin exposure led to a significant decrease (Figure 5).

Following exposure to higher concentrations of drugs (5× Cmax and 10× Cmax), only ampicillin-treated cells showed an increase in EROD activity in medium of more than 70% (Cmax: 3.63; 5× Cmax: 6.057; 10× Cmax: 6.29 pmol/L) and in plasma of about 65% (Cmax: 3.5; 5× Cmax: 5.911; 10× Cmax: 5.78 pmol/L). However, the various EROD levels of ampicillin-treated cells were still decreased compared to the medium control (8.7 pmol/L). Compared to the plasma control, the EROD levels were slightly higher (5× Cmax, 10× Cmax). The other antibiotics did not result in a significant decrease.

### 3.5. Tigecycline Led to a Noticeable Dose-Dependent Impairment of Albumin-Synthesis

We observed a significant increase in albumin synthesis after incubation at Cmax with ampicillin, cefepime, linezolid, and meropenem (Table 1). In contrast, incubation with cefuroxime, tigecycline, and vancomycin at Cmax led to a significant decrease in albumin synthesis in medium (Table 1, first column; Cmax). Furthermore, a dose-dependent decrease in albumin was observed after incubation with all tested antibiotics in medium. Synthesis of albumin was decreased to 100% compared to the control groups at higher concentrations of tigecycline in medium (Table 1, second and third columns; 5× Cmax and 10× Cmax).

In addition, in plasma, cefuroxime, meropenem, tigecycline, and vancomycin influenced negative microalbumin synthesis at the Cmax concentrations. Treatment with ampicillin, cefepime, cefuroxime, levofloxacin, linezolid, and rifampicin increased the microalbumin synthesis of the hepatocytes (Table 1, second column). However, higher concentrations of ampicillin, cefuroxime, linezolid, tigecycline, and rifampicin impaired the synthesis of albumin in test cells (Table 1, second and third columns).

The parameters used to estimate the hepatotoxic potential of the tested antibiotics at therapeutic concentrations (Cmax) are listed in Table 2. The number of stars represents the degree of hepatotoxicity in terms of impairment parameters and in relation to the negative control without specific ranking of the parameters.

## 4. Discussion

The majority of classes of antibiotics have hepatotoxic potential and are considered a leading cause of DILI [15,45]. With an established in vitro cytotoxicity screening model based on HepG2/C3A cells, we investigated the in vitro hepatotoxicity of antibiotics at clinically relevant concentrations (Cmax, 5× Cmax, and 10× Cmax) [46,47,48,49,50] used for systemic or severe infections [24,25,27,28]. To evaluate the influence of plasma protein binding on the cytotoxicity of the investigated drugs, we tested all antibiotics in plasma from healthy volunteers (pooled plasma), as well as in cell culture medium (with 10% FBS). Biologically relevant endpoints were the viability (XTT test, LDH release, and trypan blue staining) and functionality (EROD activity and synthesis of microalbumin) of the antibiotic-treated hepatocytes.

Prediction of drug-induced hepatotoxicity in humans from in vitro data remains a significant challenge [51]. First, fresh primary isolated hepatocytes are a valuable in vitro model for drug metabolism studies, representing the gold standard. However, these cells are limited due to their irregular availability; inability to multiply; and donor variability, which limits their utility. Generally, conventional in vitro immortalized human hepatocarcinoma cell lines, such as HepG2/C3A, have been used as an alternative to fresh primary isolated hepatocytes due to their availability, their unlimited lifespan, their stable phenotype, and their ease of use [52]. The biosynthetic capabilities of C3A, similar to primary human hepatocytes, such as the production of many liver specific plasma proteins [44], the presence of a functional cytochrome P450 toxin-processing enzyme system after stimulation [53,54], and glucuronic- and sulfate-conjugation abilities, were investigated and confirmed by independent investigators. Therefore, the cell line has the ability to carry out normal biotransformation reactions essential for the detoxification process. The C3A cell line does not completely mimic the behavior of liver lobules; nevertheless, C3A cells are a useful model for the predictive modeling of hepatotoxic agents [54], microfluidic devices [53], metabolomes, and tissue engineering approaches, including 3D spheroid formation [55] and cell therapeutic applications, such as the extracorporeal liver assist device (ELAD) systems [56]. Despite known metabolic limitations, guidelines for hepatotoxicity testing generally do not preclude the use of HepG2 cells and therefore remain an attractive tool for researchers [57,58]. Flynn and Ferguson [59] and Lui et al. [59] treated HepG2/C3A cells with single purified compounds and used a large number of biologically relevant assays to estimate the hepatotoxic potential of chemicals. Currently, no models exist that allow for a better forecast.

The discovery of antibiotics as a cure for bacterial infections represents a considerable advance for medicine. However, in recent decades, it has also become clear that there is no ideal antibiotic with exclusive effect on the disease-causing microorganism and that every antibiotic therapy is associated with certain risks. Collectively, many classes of antibiotics are a major cause of drug-induced liver injury (DILI) [60]. Toxic effects are known to be associated with most antibiotics, sometimes with serious consequences. Antibiotic-induced hepatotoxicity is a rare and serious event. Most cases are idiosyncratic (the adverse reaction cannot be predicted based on the drug’s pharmacological profile or preclinical toxicology tests) and occur via an immunological reaction or in response to the presence of hepatotoxic metabolites [15]. However, acute antibiotic-induced DILI is difficult to diagnose because the course of therapy is usually short, and other confounding factors are often present. In addition to the broad clinicopathological spectrum of hepatotoxicity associated with antimicrobial agents, the infectious disease being treated may itself be associated with liver dysfunction and jaundice.

For the study of drug metabolism, there is no single approach that provides all the necessary information for complete understanding; therefore, it is necessary to verify whether the conclusions derived from in vitro experiments actually correspond to the events occurring in vivo. In this discussion, we attempt to harmonize the approaches with the information from various studies to enable a better evaluation of the results. An important element in assessing causality in drug-induced liver injury is whether the implicated agent is known to cause hepatotoxicity. In a review by Einar S. Björnsson and Jay H. Hoofnagle (2015), various drugs with potential hepatotoxicity were categorized [61]. In the reported framework, drugs were categorized based on the number of published reports of convincingly documented, clinically apparent, idiosyncratic liver injury. Drugs described on the LiverTox website (http://livertox.nih.gov) were classified into five categories based on the number of published cases (category A, ≥50; category B, 12–49; category C, 4–11; category D, 1–3; category E, none).

According to some statistics, antibiotics are the second most hepatotoxic class of drugs [62]. According to unpublished data from the Medical University of Graz (1997–2007), antibiotics represent the most common drug-related cause of liver biopsies. Antibiotics and other substances can either stimulate (e.g., rifampicin, antiepileptic drugs, and alcohol) or inhibit (e.g., macrolides, antifungals, and protease inhibitors) phase I enzymes (cytochrome P450) of hepatic detoxification. In both cases, toxic metabolites may be formed, leading to direct metabolic or immunological liver damage [63].

According to the in vitro results of the antibiotics tested in this study, rifampicin, tigecycline, and vancomycin have the highest hepatotoxic potential; furthermore, rifampicin and cefuroxime at higher concentration led to a destruction of cell integrity.

Only rifampicin and cefuroxime led to a dose-dependent decrease in vitality and cell-count (proliferation) in medium and plasma. Additionally, incubation of cefuroxime at higher concentrations with the hepatoma cell line resulted in a significant increase in lactate dehydrogenase (LDH) in the cell culture supernatant. Cefuroxime is a second-generation cephalosporin. It is very rarely known to cause DILI [64,65]. Only case reports have been reported in the literature in which cefuroxime has been associated with hepatocellular-cholestatic hepatitis or mostly with cholestatic DILI [66,67]. Although the exact mechanism of cholestatic and hepatocellular DILI of cefuroxime is unknown, it is likely idiosyncratic in nature and a result of hypersensitivity. The results of our in vitro study indicate that higher concentrations of cefuroxime, for instance by accumulation, may cause destruction of cell integrity in hepatocytes; however, at the normal therapeutic Cmax concentration, no impairment of vitality or proliferation was observed. Rifampicin, an often-used ansamycin antibiotic, has been associated with more than 50 cases of liver injury in published literature [61]. It can cause idiosyncratic DILI and, rarely, intrinsic DILI. What can explain our in vitro findings of a dose-dependent decrease in proliferation and vitality? Transient abnormalities in liver function are common in the initial phase of rifampicin therapy. However, in some cases, rifampicin can cause severe hepatotoxicity, especially in patients with pre-existing liver disease. The mechanism of rifampicin hepatotoxicity is not well-characterized, but it is known that it is extensively metabolized by the liver and is a potent inducer of numerous liver enzymes, including CYP 1A2, 2C9, 2C19, and 3A4, which are responsible for drug metabolism [68,69,70]. Therefore, the cause of injury is likely metabolites that are either directly toxic or elicit an immunologic response [68,70,71].

The activity of mitochondrial dehydrogenases (XTT-test) of HepG2/C3A cells, as a basic functional test, was significantly decreased after incubation with therapeutic concentrations (Cmax) of levofloxacin, rifampicin, tigecycline, and vancomycin. A negative dose-dependent effect on mitochondrial activity was only observed for cefepime at higher concentrations (but with an increase at Cmax). Vancomycin therapy has been widely associated with hypersensitivity reactions that may be associated with mild liver injury but rarely with severe or life-threatening liver injury. These include Stevens–Johnson syndrome, toxic epidermal necrolysis, and the characteristic syndrome of drug reaction, eosinophilia, as well as systemic symptoms [72]. Liver function is usually not considered when selecting a vancomycin dosing strategy. In addition, little data support the impact of liver dysfunction on the pharmacokinetics of vancomycin [73,74,75]; an impairment of mitochondrial function has not been described until now, and in terms of potential hepatotoxicity, vancomycin was classified as a category C antibiotic [61]. In contrast, tigecycline is known to cause mitochondrial dysfunction. Tigecycline-induced liver enzyme elevation occurs more frequently than cholestatic liver injury [76]. The main mechanism by which tigecycline provokes cholestatic liver injury is unclear. Presumably, it may be related to the pharmacokinetics and esterification of tigecycline [77]. Tigecycline increases hepatic fatty acid uptake and esterification in mice and induces steatosis [78]. Pessayre also reported that tetracycline and the various tetracycline derivatives can cause extensive microvesicular steatosis of the liver by inhibiting mitochondrial respiration and β-oxidation [79]. Side effects were observed at a higher frequency in the high-dose group than in the approved-dose group [80,81]. Tigecycline is metabolized and eliminated primarily by the liver. Liver failure induced by critical illness may have a profound effect on the pharmacokinetics of tigecycline [82]. Generally, tigecycline does not require dose adjustment in patients with mild to moderate liver problems. However, in patients with severe liver problems, the dose should be reduced and closely monitored [77]. In addition, levofloxacin use can also be associated with impairment of mitochondrial function. Levofloxacin can cause hepatotoxicity in rare cases, including cases of liver failure [83,84]. Patients with pre-existing liver damage may be particularly susceptible; in such cases, levofloxacin would not be the best choice. Fluoroquinolone antibiotics at clinically relevant concentrations have been shown to cause mitochondrial dysfunction through the production of reactive oxygen species [85]. Mitochondrial damage has also been described in hepatitis and cirrhosis as a cause of extensive liver injury [86,87]. Owing to its widespread use, levofloxacin has been associated with at least 50 cases of clinically apparent liver injury, mostly in single case reports, and has therefore been grouped in category B [88]. A relationship between the use of cefepime and impairment of mitochondrial functions has not been described. The results of our in vitro investigations show that higher concentrations of cefepime, for instance by accumulation, may cause a decrease in the activity of mitochondrial dehydrogenases in hepatocytes; however, at the normal therapeutic Cmax concentration, no impairment was observed. Although cefepime-induced neurotoxicity and nephrotoxicity have been reported in recent years, there are currently no formal reports of hepatic injury caused by this drug [89]. Cefepime is assigned a low probability value (category D) according to a review by Einar S. Björnsson and Jay H. Hoofnagle [61], with respect to causing clinically visible liver damage.

The cytochrome (P450) 1A2 enzyme (CYP 1A2) is most important for the metabolism of foreign substances. In our in vitro study, we observed a significant increase in CYP 1A2 activity after incubation with levofloxacin and linezolid at therapeutic concentrations (Cmax), whereas ampicillin, cefepime, cefuroxime, meropenem, rifampicin, tigecycline, and vancomycin led to a significant decrease. At higher concentrations of the tested drugs (5× Cmax and 10× Cmax), only ampicillin-treated cells showed a slight increase in CYP 1A2 activity in the range of the cell-culture control. The decrease in CYP 1A2 activity after incubation with levofloxacin and linezolid in vitro is surprising, as neither substance is known as a CYP 1A2 inducer or substrate [90,91,92]. In addition, rifampicin is described as an inducer of CYP 1A2 [93], although in our model, we observed a decrease. Additionally, the observed decrease in CYP 1A2 after incubation with ampicillin, cefepime, cefuroxime, meropenem, tigecycline, and vancomycin has not been previously described in the literature [94,95,96,97]. For our experiments, the test cells were stimulated before incubation with the tested antibiotics with the induction reagent β-naphthoflavone (BNF), which was added to the hepatocytes 72 h before treatment and measurement. In our own previous experiments and in the literature, a pretreatment of hepatocytes with BNF over 72 h was validated. In a recent paper, Lněničková et al. described an inducer variability over time and recommended kinetic measurements depending on the measured parameter [98].

The production of proteins, such as albumin, is an important function of the liver. In our in vitro model, we found that incubation with cefuroxime, tigecycline, and vancomycin at Cmax led to a significant decrease in albumin synthesis in medium. Furthermore, a dose-dependent decrease in albumin was determined after incubation with all antibiotics in medium; however, after incubation with the 10× Cmax concentration of tigecycline, a complete breakdown of albumin synthesis was observed. These are new findings, and the mechanisms are currently unclear. Treatment with tigecycline can lead to inhibition of mitochondrial respiration and β-oxidation [79]. Under inflammatory conditions, a deficit of antioxidants led to an inhibition of negative acute-phase protein secretion (e.g., albumin), which may also lead to toxic conditions [99].

The used “broad-spectrum” hepatotoxicity test was previously validated in some clinical and experimental studies [24,25,26,27,28]. Table 2 summarizes the six parameters used to estimate the hepatotoxic potential of the tested antibiotics at therapeutic concentrations (Cmax). All tested antibiotics showed minimum impairment in two parameters. These findings were evaluated as mild hepatotoxicity; drugs with more than three positive impairment parameters were evaluated as moderate hepatotoxicants (rifampicin, tigecycline, and vancomycin).

The antibiotics meropenem, linezolid, and ampicillin should be also discussed. Meropenem often causes only mild, transient elevations in aminotransferase and rarely causes clinically apparent cholestatic liver damage [100]. The cause of mild, transient elevations in serum enzymes during meropenem therapy is unknown. Cholestatic hepatitis attributable to carbapenems is probably immunoallergic in nature and resembles the rare clinically visible liver injury that has been associated with penicillin and cephalosporins. In our experiments, meropenem at a therapeutic concentration (Cmax) led to an increase in lactate dehydrogenase and a decrease in CYP 1A2 and mitochondrial dehydrogenase activity (XTT test, only in plasma). Due to the low incidence of hepatotoxicity identified in association with meropenem, this antibiotic was classified as category D [61]. Linezolid is a category C drug according to a review by Einar S. Björnsson and Jay H. Hoofnagle [101]. It has been hypothesized that linezolid inhibits the human mitochondrial ribosome by a similar mechanism due to the similarities between human and bacterial ribosomes [102]. As a result, the lack of mitochondrially encoded proteins has been associated with rare cases of lactic acidosis and liver damage, likely due to hepatic mitochondrial toxicity [103,104,105]. In our investigations, incubation with linezolid with the hepatoma cell line at the Cmax concentration led to a decrease in vitality and cell count [106]. Ampicillin is a third-generation oral penicillin that is among the most frequently used antibiotics worldwide. It is commonly used to treat mild to severe infections and has been associated with idiosyncratic liver injury at a very low rate and predominantly in single case reports. In our analyses, we confirmed its low hepatotoxic potential [103]; an increase in lactate dehydrogenase in cell culture supernatant and reduced CYP 1A2 activity were also observed. In ampicillin-related hepatotoxicity, the damage is mainly hepatocellular. The serum enzyme pattern associated with aminopenicillin liver injury includes a hepatocellular pattern, with marked elevations of transaminases, minimal elevations of alkaline phosphatase, and rapid recovery after discontinuation of antibiotic treatment. Cholestatic forms of liver injury with marked elevations of alkaline phosphatase have also been described [107]. Ampicillin alone was classified as category C by the working groups by Einar S. Björnsson and Jay H. Hoofnagle [61]. Under clinical conditions, aminopenicillins ampicillin and amoxicillin are often administered in combination with ß-lactamase inhibitors clavulanate and sulbactam. Especially clavulanate, but also sulbactam, has a high and well-known hepatotoxic potential [107,108,109].

## 5. Conclusions

It is difficult to identify the action of single hepatotoxic drugs because treatment is often administered in combination with many other drugs, and most antibiotics can cause hepatotoxic reactions. In our experiments, we tested antibiotics that are frequently use in intensive care units. Rifampicin, tigecycline, and vancomycin are the most commonly used drugs with high hepatotoxic potential; therefore, they should be used with caution in patients with pre-existing liver disease/insufficiency (summarized in Table 2). Conversely, the other tested antibiotics, ampicillin, cefepime, cefuroxime, levofloxacin, linezolid, and meropenem showed only a mild hepatotoxic potential in vitro. Rifampicin and cefuroxime showed markedly negative effects on viability of the human hepatoma cell line.

Further in vitro studies and global pharmacovigilance reports should be conducted to reveal the underlying mechanism of the hepatotoxic action of vancomycin, rifampicin, tigecycline, and cefuroxime, as well as the clinical relevance of these findings. Unfortunately, recognition of the toxicity of certain drugs is limited by inadequate reporting and the relatively rare overall frequency of hepatotoxicity. Diligent reporting in DILI cases registries [19,20,21,22,110] could improve the database of cases of hepatotoxicity caused by antibiotics. In addition, improved in vitro models may help to improve understanding of the exact mechanisms of hepatotoxicity. There is a lack of knowledge about some aspects and molecular biological mechanisms of antibiotic-induced hepatotoxicity. Current developments with respect to improved in vitro models include microfluidic models, 3D models using different kinds of cells, and the use of induced pluripotent stem (iPS) cells [111,112,113,114].

Careful drug monitoring is particularly important in patients with a history of hepatotoxic reactions to antibiotics, the elderly, and patients with impaired liver function. Due to the increase in old and sick patients in intensive care units, as well as the increasing tendency of predamaged livers, the risk–benefit ratio of antibiotic therapy should be maximized.

## Figures and Tables

**Table 1:** Overview of the microalbumin (MA) levels of antibiotic-treated C3A cells in medium (first column) and in plasma (second column) at all tested concentrations. Values represent median and 25th/75th percentile. * indicates a significance level of *p* < 0.05 relative to the negative control.

Parameter	Microalbumin [mg/L]. Medium			Microalbumin [mg/L]. Plasma		
Testsubstance	Cmax	5x Cmax	10x Cmax	Cmax	5x Cmax	10x Cmax
**Ampicillin**	9.37 * 8.64/9.96	5.21 * 5.01/5.41	4.66 * 4.56/4.84	57.65 * 55.83/62.43	**31.65** 18.00/38.43	**30.15** 23.35/48.93
**Cefepim**	9.23 * 6.93/12.15	**5.69** 4.99/7.83	5.44 * 4.78/6.63	94.90 * 80.90/106.00	85.15 * 72.88/106.80	96.90 * 85.40/109.80
**Cefuroxime**	4.26 * 2.31/5.01	1.01 * 0.00/2.77	2.16 * 0.00/2.54	6.73 * 5.33/7.67	5.71 * 5.31/6.63	5.88 * 5.23/10.26
**Levofloxacin**	**8.55** 6.43/11.93	13.45 * 11.98/20.15	11.30 * 10.30/14.10	134.50 * 99.58/146.80	129.00 * 101.00/135.80	98.10 * 36.95/147.50
**Linezolid**	8.87 * 7.85/11.02	**6.47** 5.71/8.91	4.35 * 3.03/5.97	90.95 * 86.15/108.20	94.05 * 79.75/104.70	73.80 * 59.98/87.65
**Meropenem**	9.68 * 8.87/11.60	5.43 * 4.83/7.78	4.97 * 4.00/6.17	**21.40** 11.58/65.65	**17.80** 10.15/78.20	**24.90** 14.25/66.70
**Rifampicin**	**7.49** 5.30/12.43	**7.49** 5.30/12.45	**6.48** 0.00/8.35	106.00 * 34.78/136.00	32.60 * 19.08/102.50	38.65 * 14.95/99.00
**Tigecyline**	5.57 * 4.48/6.99	2.86 * 0.55/3.05	0.00 * 0.00/0.00	**27.95** 14.10/60.05	**35.15** 11.43/68.02	**20.85** 6.76/48.50
**Vancomycin**	**7.28** 6.35/8.57	5.99 * 5.04/6.93	**6.61** 4.99/7.15	**10.45** 8.96/11.55	**9.97** 7.81/13.80	**12.30** 10.28/13.68
pos.Ctrl	0.00 0.00/3.15			2.96 3.73/4.95		
neg. Ctrl	6.59 5.68/8.78			20.40 7.93/50.70		

**Table 2:** Comparison of Cmax of the tested antibiotics in medium (M) and in plasma (P) compared to the negative control (neg. Ctrl.). The number of stars (significant difference relative to the negative control) represents the hepatotoxic potential of the tested antibiotics in medium and plasma at clinically relevant concentrations. MA: microalbumin.

	neg. Ctrl		Ampicillin		Cefepime		Cefuroxime		Levofloxacin		Linezolid		Meropenem		Rifampicin		Tigecycline		Vancomycin	
Protein binding [%]			20		20		50–70		30–40		30		15		75–80		90		50	
**Medium/Plasma**	M	P	M	P	M	P	M	P	M	P	M	P	M	P	M	P	M	P	M	P
**Cell Count (×100,000)**	**7.8**	**5.6**							↓		↓				↓		↓	↓		
**Vitality [%]**	**94**	**91**							↓	↓	↓	↓							↓	
**LDH 6d [U/L]**	**91**	**168**	↑		↑		↑						↑		↑		↑		↑	
**XTT (OD)**	**1.38**	**1.26**							↓	↓				↓	↓		↓	↓	↓	↓
**MA [mg/L]**	**6.6**	**33.1**					↓	↓									↓	↓		↓
**CYP1A2 [pmol/L]**	**8.77**	**5.3**	↓	↓	↓	↓	↓	↓					↓	↓	↓	↓	↓	↓	↓	↓
**Hepatotoxic-Potential**			**	*	**	*	***	**	***	**	**	*	**	**	****	*	*****	****	****	***

Arrow and up arrow: Increase and Decrease compared with the control.

## Data Availability

The data presented in this study are available upon request from the corresponding author.
